# In Situ Dynamic Spectroscopic Ellipsometry Characterization of Cu-Ligated Mercaptoalkanoic Acid “Molecular” Ruler Multilayers

**DOI:** 10.3390/mi15070826

**Published:** 2024-06-26

**Authors:** Alexandra M. Patron, Kayleigh L. Coleman, Thomas J. Mullen

**Affiliations:** Department of Chemistry and Biochemistry, University of North Florida, Jacksonville, FL 32224, USA

**Keywords:** spectroscopic ellipsometry, metal-ligated multilayer, molecular ruler, hybrid nanolithography, mercaptohexadecanoic acid, self-assembled monolayer

## Abstract

Hybrid strategies that combine conventional top-down lithography with bottom-up molecular assembly are of interest for a range of applications including nanolithography and sensors. Interest in these strategies stems from the ability to create complex architectures over large areas with molecular-scale control and precision. The molecular-ruler process typifies this approach where the sequential layer-by-layer assembly of mercaptoalkanoic acid molecules and metal ions are combined with conventional top-down lithography to create precise, registered nanogaps. However, the quality of the metal-ligated mercaptoalkanoic acid multilayer is a critical characteristic in generating reproducible and robust nanoscale structures via the molecular-ruler process. Therefore, we explore the assembly of alkanethiolate monolayers, mercaptohexadecanoic acid (MHDA) monolayers, and Cu-ligated MHDA multilayers on Au{111} substrates using atomic force microscopy and in situ dynamic spectroscopic ellipsometry. The chemical film thicknesses in situ dynamic spectroscopic ellipsometry agree with previous ex situ surface analytical methods. Moreover, in situ dynamic spectroscopic ellipsometry provides insight into the assembly process without interrupting the assembly process and potentially altering the characteristics of the resulting chemical film. By following the real-time dynamics of each deposition step, the assembly of the Cu-ligated MHDA multilayers can be optimized to minimize deposition time while having minimal impact to the quality of the chemical film.

## 1. Introduction

Tremendous effort has been devoted to production of nanoscale structures and devices with tailored chemical functionalities for a broad range of applications beyond traditional semiconductor manufacturing [1,2,3]. These efforts have been used for a number of applications including molecular resists for lithography and regulating the growth of polymers and biomolecules for sensors [4,5]. Hybrid strategies that combine conventional top-down lithography with bottom-up molecular assembly are of particular interest towards these efforts because they enable the creation of complex architectures over large areas with molecular-scale control of the chemical and physical properties [1,2].

The molecular-ruler process exemplifies this combination of conventional top-down patterning with molecular-scale assembly as means to create precise nanogaps (4–100 nm) between lithographically defined metal surface structures [6,7,8,9,10,11,12]. In the first steps of this process, metal structures are patterned across a semiconductor substrate using conventional lithography. A metal-ligated multilayer is assembled on the metal structures through the iterative deposition of bifunctional organic molecules and metal ions. The thickness of the multilayer is governed by the number of deposition steps of sequential alternating layers. Typically, mercaptoalkanoic acid molecules are used in combination with Cu(ClO_4_)·6H_2_O as the mercaptoalkanoic acid molecules preferentially adsorb to the metal structures and the Cu(II) ions preferentially adsorb to the mercaptoalkanoic acid molecules. Once the desired thickness of the multilayer is achieved, a second metal is deposited across the substrate. Subsequently, a chemical lift-off removes the labile multilayer exposing the initial metal and the regions of the substrate masked by multilayer. This yields a nanogap between the two metal structures with the dimensions of the thickness of the multilayer. The molecular-ruler process provides a general and widely applicable hybrid methodology that enables the creation of nanometer-scale registered features for applications ranging from nanoelectronics to electrochemical sensors [13,14,15,16].

The quality of the Cu-ligated mercaptoalkanoic acid multilayers remain a critical characteristic in generating reproducible and robust nanoscale structures via the molecular-ruler process. Therefore, considerable effort has been devoted to better understand the assembly, structure, and characteristics of Cu-ligated mercaptoalkanoic acid multilayers [17,18,19,20,21,22]. However, most, if not all, of these studies rely on ex situ characterization of the Cu-ligated mercaptoalkanoic acid multilayers where the assembly process must be interrupted because the substrates must be rinsed and dried prior to characterization. While these ensemble and local surface characterization strategies provide valuable insights into the structure and quality of a Cu-ligated mercaptoalkanoic acid multilayer, the real-time dynamics of the assembly of the multilayer is generally lost, making it challenging and time consuming to optimize the conditions (e.g., solution concentrations, deposition time, etc.) for multilayer assembly. Furthermore, the assembly of subsequent layers could potentially be influenced by multiple rinsing and drying steps required to follow the multilayer assembly process. Anderson and co-workers circumvented the latter limitation by creating sample sets of successive layers by cleaving a portion of a larger substrate for characterization and then using the remainder of the substrate to continue multilayer growth [21]. However, there is a limited number of substrates that can be created using this elegant approach, and these substrates still rely on ex situ characterization. Therefore, it is vital that in situ characterization strategies be developed so as to mitigate these limitations and provide further insight in the Cu-ligated mercaptoalkanoic acid multilayer assembly process.

In this report, we set out to explore the capabilities of in situ dynamic spectroscopic ellipsometry to study the assembly and growth of alkanethiolate monolayers, mercaptohexadeconic acid (MHDA) monolayers, and Cu-ligated MHDA multilayers on Au{111} substrates. Cu-litigated MHDA multilayers are the most common multilayers used in the molecular-ruler process and have been shown to produce high-quality, robust, and consistent chemical multilayers [17,19,20,21,22]. Specifically, the assembly of alkanethiolate monolayers on Au{111} substrates are followed using in situ dynamic spectroscopic ellipsometry to demonstrate the utility of this technique [23]. These chemical films have been extensively studied using a range of surface analytical techniques and produce highly ordered monomolecular films. Subsequently, MHDA monolayers and Cu-ligated MHDA multilayers comprised of up to four layers of MHDA molecules are characterized using atomic force microscopy (AFM) and in situ dynamic spectroscopic ellipsometry. AFM is ideally suited to confirm the local structure and morphology of MHDA monolayers and Cu-ligated MHDA multilayers fabricated on Au{111} substrates. In situ dynamic spectroscopic ellipsometry follows the multilayer assembly process in real time and demonstrates the utility of the characterization method.

## 2. Materials and Methods

### 2.1. Reagents and Materials

All reagents were used as received. 1-dodecanethiol (C12, 95%), 1-octadecanethiol (C18, 95%), 16-mercaptohexadeconic acid (MHDA, 90%), copper(II) perchlorate hexahydrate (Cu(ClO_4_)·6H_2_O, 98%), acetic acid (>99%), and n-ocadecyltrichlorsilane (OTS, >90%) were purchased from Sigma Aldrich (St. Louis, MO, USA). Sulfuric acid (ACS grade), hydrogen peroxide (30% aqueous solution), chloroform (HPLC grade), and hexadecane (>98%) were purchased from VWR International (Randor, PA, USA). Absolute ethanol was purchased from Pharmco-Aaper (Brookfield, CT, USA). Au pellets (99.999%) were purchased from Kurt J. Lesker Company (Pittsburgh, PA, USA). Ruby muscovite mica (Ruby Mica Optical Grade V-2) was purchased from S&J Trading Inc. (Floral Park, NY, USA). 18 MΩ water was created using a Milli-Q system (Q-GARD 2, Billerica, MA, USA). All glassware was cleaned by immersing in piranha solution (3:1 by volume of sulfuric acid/30% hydrogen peroxide) for 1 h, rinsing with copious amounts of 18 MΩ water, and drying overnight. Caution: piranha solution is a vigorous oxidant and should be handled with extreme care!

### 2.2. Fabrication of Au Substrates

Au{111} substrates were prepared following previous work [24,25]. Freshy-cleaved mica substrates were placed into a custom-built thermal deposition system. After reaching a pressure of <10^−6^ torr, the mica was heated to 350 °C and 150–200 nm of Au was deposited at a rate of 0.1–0.2 nm/s. Subsequently, the Au substrates were annealed at 350 °C for 30 min, allowed to cool to room temperature, and removed from the thermal deposition system. Prior to their use, the Au{111} substrates were annealed using a hydrogen flame for 30 s.

### 2.3. Atomic Force Microscopy Characterization and Analysis

Contact-mode AFM images were acquired with a Keysight 5420 scanning probe microscope using sharpened Si_3_N_4_ cantilevers (DNP-S, Buker AFM Probes, Santa Barbara, CA, USA) with nominal force constants of 0.35 N/m. Force constants for individual cantilevers were determined using the thermal noise method [26]. The cantilevers were cleaned using a benchtop UV ozone tool (Novascan, PSDP-UVT, Ames, IA, USA) for 45 min, immersed into 1 mM OTS solution (7:3 mixture of hexadecane and chloroform) for 1 h, rinsed with chloroform, and dried under a stream of N_2_. This cleaning process removed surface contaminants from the Si_3_N_4_ cantilevers and minimized the adsorption of MHDA molecules to the Si_3_N_4_ cantilevers during imaging [27,28,29]. Force setpoints for imaging of ~1 nN were used to minimize disruption to the MHDA monolayers and Cu-ligated MHDA multilayers. AFM images were acquired at 256 points per line at a scan rate of 1 Hz and under ethanol to minimize surface contamination. Image processing and analysis of the AFM images were performed using Gwyddion (version 2.65, “Arithmetic Amends”); this open-source software is freely available on the internet and is supported by the Czech Metrology Institute [30]. The average and standard deviation of the root-mean-squared (RMS) roughnesses for the MHDA monolayers and Cu-ligated MHDA multilayers were calculated from 250 nm × 250 nm squares in at least five locations of each type of chemical film.

### 2.4. Spectroscopic Ellipsometry

Spectroscopic ellipsometry measurements were acquired using a rotating compensator spectroscopic ellipsometer (Alpha-SE, J.A. Woollam Inc., Lincoln, NE, USA) where 180 wavelengths between 380 and 900 nm were measured at a fixed 70° angle of incidence. All measurements were acquired using a liquid cell (500 µL Alpha Liquid Cell, J.A. Woollam Inc., Lincoln, NE, USA) under ethanolic solutions. All solutions were flushed through the liquid cell using a peristaltic pump (Mini-Pump Variable Flow, VWR International, Randor, PA, USA) with a flow rate of 20 mL/min. Dynamic ellipsometry measurements were acquired every 11 s throughout the duration of each experiment. The thicknesses of the MHDA monolayers and Cu-ligated multilayers were calculated using the CompleteEASE Software package (version 6.70). A B-spline model was used for the Au substrates, and a Cauchy model with a refractive index of *n* = 1.5 was used for the C12/C18/MHDA monolayers and Cu-ligated MHDA multilayers [12,17,20,21]. The ambient optical constants for absolute ethanol were automatically applied to all fits and were determined by measuring the optical constants of an ethanol solution atop a glass slide with roughed backside to suppress backside reflation and fitting them to a Cauchy model. The optical contributions of the liquid cell windows were accounted for by measuring the optical constants of a Au substrate mounted in the liquid cell prior to being flushed with ethanol. The average and standard deviation of the resulting calculated thickness were determined from 10 measurements for each deposition step.

### 2.5. Preparation of Alkanethiolate Monolayers, MHDA Monolayers, and Cu-Ligated MHDA Multilayers

**Figure 1:**
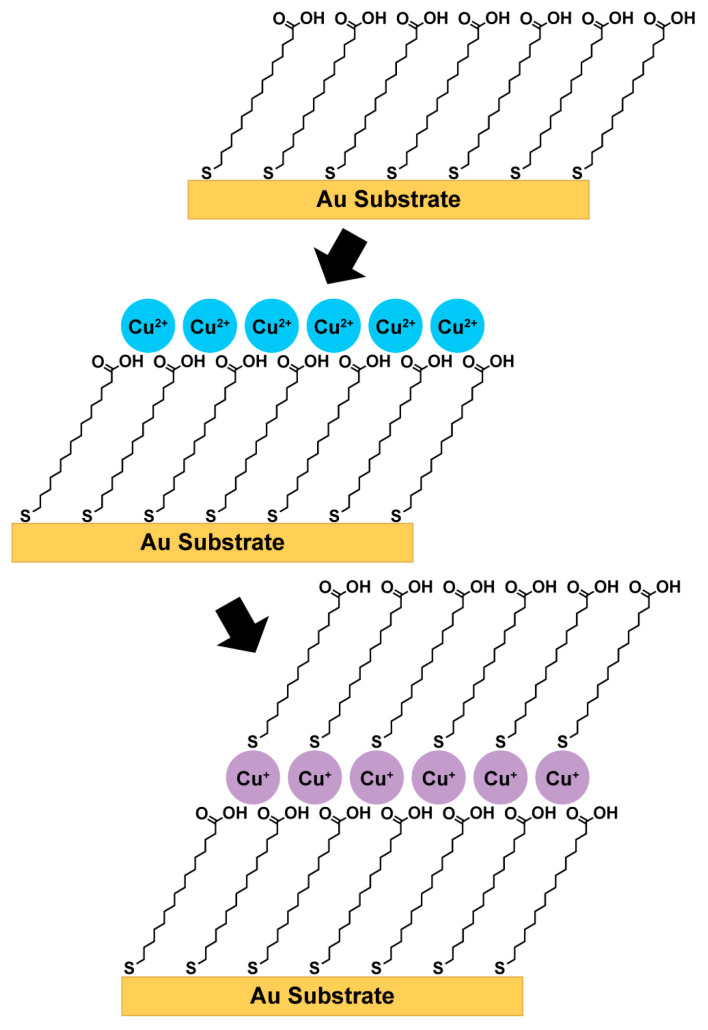
General approach for the fabrication of Cu-ligated MHDA multilayers on Au{111} substrates. MHDA monolayers are formed by exposing Au{111} substrates to 0.01 mM MHDA and 1.5 M acetic acid ethanolic solutions. The Cu-ligated MHDA multilayers are assembled via sequential immersion ethanolic solutions of 5 mM Cu(ClO_4_)_2_·6H_2_O and 1 mM MHDA until the number of desired layers is achieved.

Cu-Ligated MHDA multilayers were fabricated by the general alternating-layer-assembly strategy outlined in Figure 1. Initially, MHDA monolayers were formed by exposing Au{111} substrates to MHDA and acetic acid ethanolic solutions. Acetic acid minimized MHDA dimerization and agglomeration by competing for hydrogen bonding interactions [22,31,32,33]. Subsequently, the Au{111} substrates were removed from the MHDA and acetic acid ethanolic solutions, rinsed with ethanol, and exposed into Cu(ClO_4_)_2_·6H_2_O ethanolic solutions. The Au{111} substrate was then removed from the Cu(ClO_4_)_2_·6H_2_O ethanolic solution, rinsed with ethanol, and exposed to MHDA ethanolic solutions. This sequence of immersion into Cu(ClO_4_)_2_·6H_2_O and MHDA ethanolic solutions was repeated until the number of desired layers was achieved. It is important to note that only the initial MHDA monolayers were formed from MHDA and acetic acid ethanolic solutions; the subsequent layers of Cu-ligated MHDA multilayers were formed from ethanolic solutions without acetic acid. Acetic acid was previously shown to disrupt the assembly of the Cu-Ligated MHDA multilayers [22].

For AFM experiments, MHDA monolayers and Cu-Ligated MHDA multilayers were prepared on Au{111} substrates and subsequently imaged with contact-mode AFM. MHDA monolayers were formed by immersing Au{111} substrates into v-vials containing 0.01 mM MHDA and 1.5 M acetic acid ethanolic solutions overnight. Subsequently, the Au{111} substrates were rinsed with ethanol and dried under a stream of N_2_. Cu-ligated MHDA multilayers were formed by immersing the MHDA monolayers on Au{111} substrates into v-vials containing 5 mM Cu(ClO_4_)_2_·6H_2_O for 5 min. Upon removal from the Cu(ClO_4_)_2_·6H_2_O ethanolic solutions, the Au{111} substrates were rinsed with ethanol and dried under a stream of N_2_. Subsequently, the Au{111} substrates were immersed into v-vials containing 1 mM MHDA ethanolic solutions for 1 h, rinsed with ethanol, and dried under a stream of N_2_. This sequence of immersion into v-vials of 5 mM Cu(ClO_4_)_2_·6H_2_O and 1 mM MHDA ethanolic solutions followed by rinsing with ethanol and drying under a stream of N_2_ was repeated until the number of desired layers was achieved. All MHDA monolayers and Cu-ligated MHDA multilayers were imaged immediately after preparation.

For in situ dynamic spectroscopic ellipsometry experiments, C18, C12, and MHDA monolayers as well as Cu-ligated MHDA multilayers were formed on Au{111} substrates mounted within the 500 µL Alpha liquid cell. The liquid cell, and Au{111} substrate mounted within, was first flushed with 15 mL of ethanol in order to measure the optical properties of the clean, unfunctionalized Au{111} substrates. Based on the flow rate of 20 mL/min, 15 mL of ethanol was flushed through the liquid cell in 40–50 s. This volume and flow rate was sufficient for ethanol (or other ethanolic solutions) to bathe the Au{111} substrate, remove air bubbles in the liquid cell, and prevent cross contamination with subsequent solutions. The in situ dynamic spectroscopic ellipsometry measurements were started once the ethanol was flushed through the liquid cell and were acquired every ~11 s. This acquisition time was limited to the length of time it took to complete a single spectroscopic measurement. Ethanol was allowed to steep in the fluid cell for 5 min until the next solution was flushed through the liquid cell. To form C18 and C12 monolayers on unfunctionalized Au{111} substrates, 15 mL of 1 mM C18 (or 1 mM C12) ethanolic solutions were flushed through the liquid cell and allowed to remain in the fluid cell for 120 min. Subsequently, 15 mL of ethanol was flushed through the liquid cell and allowed to remain for 5 min.

To form MHDA monolayers on unfunctionalized Au{111} substrates, 15 mL of a 0.01 mM MHDA and 1.5 M acetic acid ethanolic solution were flushed through the liquid cell and remained in the fluid cell for 120 min. Subsequently, 15 mL of ethanol was flushed through the liquid cell and allowed to steep for 10 min. To assemble the Cu-Ligated MHDA multilayers atop the MHDA monolayers, 15 mL of a 5 mM Cu(ClO_4_)_2_·6H_2_O ethanolic solution was flushed through the fluid cell; the 5 mM Cu(ClO_4_)_2_·6H_2_O ethanolic solution stayed in the fluid cell for 10 min. Subsequently, 15 mL of ethanol was flushed through the fluid cell and allowed to steep in the fluid cell for 10 min. Next, 15 mL of a 1 mM MHDA ethanolic solution was flushed through the liquid cell and allowed to steep in the fluid for 20 min. Subsequently, 15 mL of ethanol was flushed through the liquid cell and allowed to steep in the fluid cell for 10 min. This sequence of flushing with 5 mM Cu(ClO_4_)_2_·6H_2_O and 1 mM MHDA ethanolic solutions followed by rinsing with ethanol was repeated until the number of desired layers was achieved.

## 3. Results and Discussion

### 3.1. Alkanethiolate Monolayer Formation Investigated Using In Situ Dynamic Spectroscopic Ellipsometry

**Figure 2:**
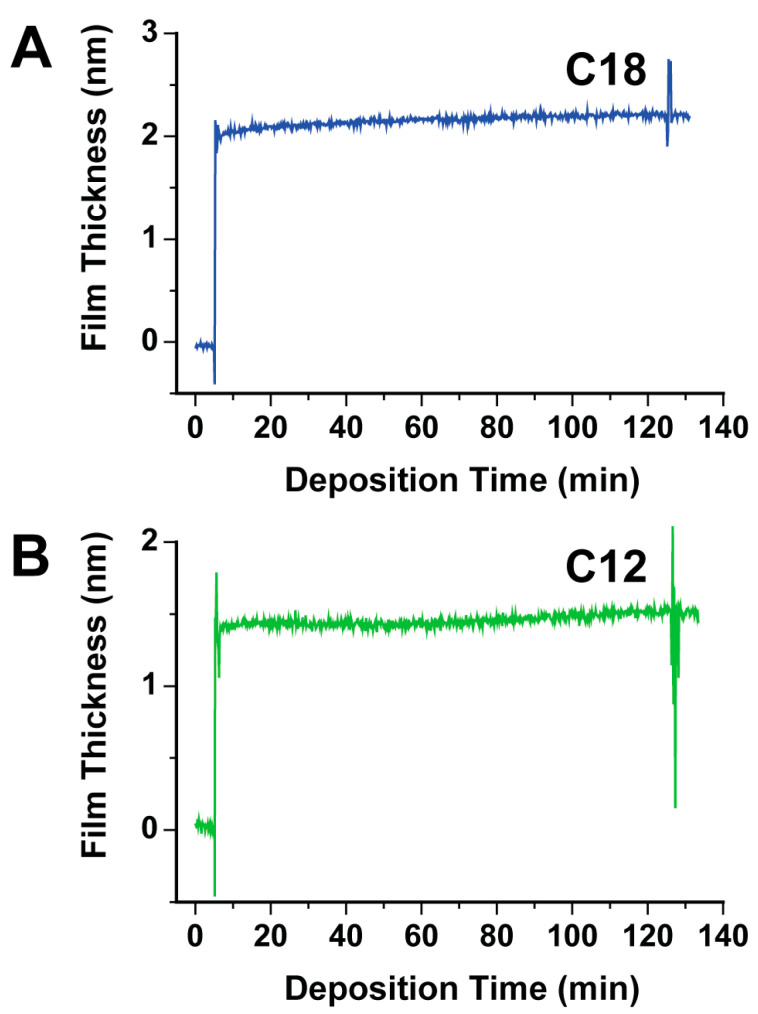
In situ Dynamic spectroscopic ellipsometry data displaying the film thickness of (**A**) a C18 monolayer and (**B**) a C12 monolayer as a function of time.

To investigate the capabilities of in situ dynamic spectroscopic ellipsometry on the assembly of alkanethiolate molecules on Au{111} substrates, the growth of C18 and C12 monolayers is followed. These molecules have been extensively studied and form highly ordered monolayers on Au{111} substrates. Figure 2A displays the film thickness as a function of deposition time during the assembly of a C18 monolayer. After the initial ethanol is flushed through the liquid cell, the film thickness measures 0.0 ± 0.1 nm, indicating a clean and unfunctionalized Au{111} substrate. When 15 mL of a 1 mM C18 ethanolic solution is flushed through the liquid cell, the film thickness rapidly increases to 1.9 ± 0.1 nm and then slowly increases to 2.2 ± 0.1 nm over 120 min. This is consistent with the alkanethiolate monolayer formation process of rapid adsorption followed by slow ordering and molecular exchange [34,35,36]. When 15 mL of ethanol is flushed through the liquid, the film thickness measures 2.2 ± 0.1 nm, which is consistent with previous theoretical and experimental values [29,33,37,38,39]. Since the in situ spectroscopic ellipsometry measurements are corrected using the ambient optical constants for absolute ethanol, the film thickness can be compared to previous theoretical and experimental values. It is important to note that we observe little to no differences in the film thicknesses between the dilute thiol ethanolic solution and absolute ethanol solution. Figure 2B shows the film thickness as a function of deposition time during the assembly of a C12 SAM. Similar features and trends are observed with the growth of a C12 monolayer when compared to the growth of a C18 SAM. When 15 mL of a 1 mM C12 ethanolic solution is flushed through the liquid cell, the film thickness rapidly increases to 1.4 ± 0.1 nm and is followed by a slow increase to 1.5 ± 0.1 nm. When 15 mL of ethanol is flushed through the liquid cell, the film thickness measures 1.5 ± 0.1 nm; again, these measurements are consistent with previous theoretical and experimental values of C12 monolayers [29,33,37,38,39].

When the liquid cell is flushed with a solution, large fluctuations in the film thickness are observed. These fluctuations in film thickness are independent of the solution composition. Once the solution is allowed to steep, the fluctuations in film thickness are no longer observed. This suggests that they are due to the disruption of the spectroscopic ellipsometry measurements during solution flow.

### 3.2. MHDA Monolayer and Cu-Ligated MHDA Multilayers Characterized Using AFM

**Figure 3:**
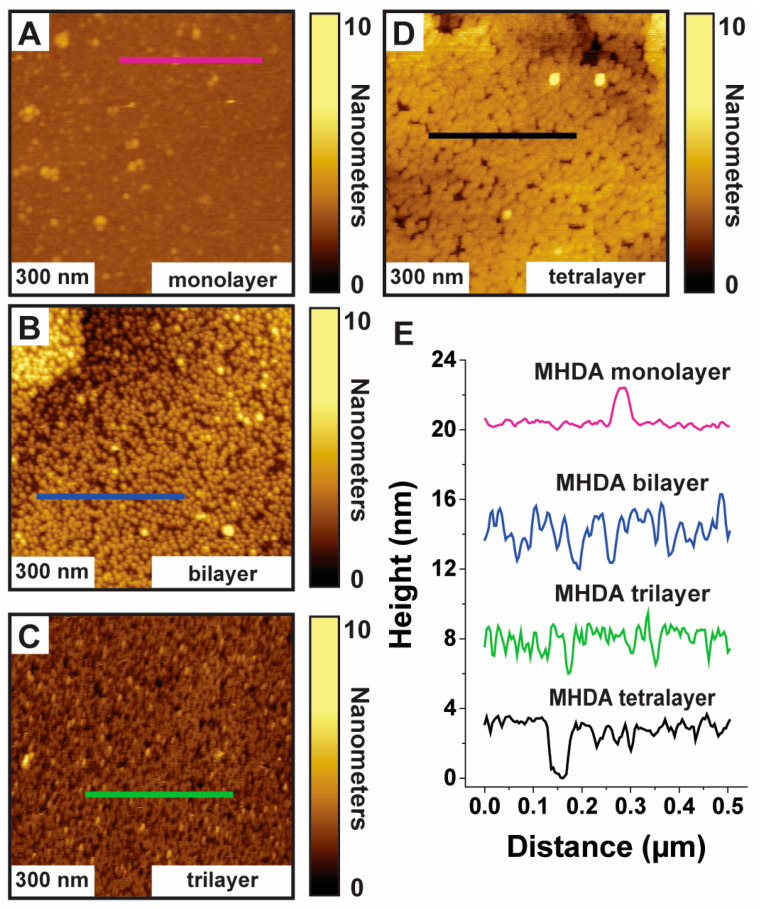
Representative AFM images of (**A**) a MHDA monolayer, (**B**) a Cu-ligated MHDA bilayer, (**C**) a Cu-ligated MHDA trilayer, and (**D**) a Cu-ligated MHDA tetralayer fabricated on Au{111} substrates. (**E**) 500 nm Cursor profiles across the substrates as indicated by the purple, blue, green, and black lines. AFM images were acquired under ethanol with force setpoints of ~1 nN and scan rates of 1 Hz.

The local morphology and structures of MHDA monolayers and Cu-ligated MHDA multilayers fabricated on Au{111} substrates are investigated using AFM. Figure 3A shows a AFM image of a 1 µm × 1 µm region of MHDA monolayer on a Au{111} substrate, and Figure 3E shows a cursor profile across the monolayers as indicated by the purple line in Figure 3A. The morphology and RMS roughness (0.3 ± 0.1 nm) of the MHDA monolayer are consistent with densely packed MHDA monolayers [19,20,21,22,29]. Figure 3B–D show AFM images of 1 µm × 1 µm regions of a Cu-ligated MHDA bilayer, trilayer, and tetralayer on Au{111} substrate, respectively. Figure 3E shows cursor profiles across the multilayers as indicated by the blue, green, and black lines in Figure 3B–D, respectively. The morphology and RMS roughness (1.2 ± 0.1 nm) of the Cu-ligated bilayer is dissimilar to the relatively smooth morphology of the MHDA monolayer. Protruding domains are observed across the surface with heights of ~2 nm and surface coverages of ~50%. This indicates an incomplete second MHDA layer atop the MHDA monolayer. The morphology of the Cu-ligated MHDA trilayer suggests a more complete multilayer when compared to the Cu-ligated bilayer as there are fewer protruding domains and a decrease in the RMS roughness (0.9 ± 0.1 nm). Similarly, the morphology and RMS roughness (0.7 ± 0.1 nm) of the Cu-ligated MHDA tetralayer indicates an even more complete multilayer when compared to the Cu-ligated MHDA trilayer. Protruding features, with heights ranging from 1 to 5 nm, are observed across the MHDA monolayer and Cu-ligated MHDA multilayers; these features are attributed to the dimerization and agglomeration of solute MHDA molecules to the carboxylate moiety of the MHDA monolayers and multilayers [19,20,22].

For the Cu-ligated MHDA trilayers and tetralayers, multilayer growth appears to occur across most of the substrate (>90%). However, for a small fraction of the substrate (<10%), depressed features, with depths of ~2 nm and ~4 nm, are observed for both the Cu-ligated MHDA trilayers and tetralayers, respectively. The 2 nm depressed features for the Cu-ligated MHDA trilayer correspond to 1-layer defects, and the 4 nm depressed features for the Cu-ligated MHDA tetralayer correspond to 2-layer defects. This suggests that although the Cu-ligated MHDA multilayers grow across most of the surface, there are a few regions where the Cu-ligated multilayer does not grow after the 2nd layer. These variations of Cu-ligated multilayers thickness due to the termination of multilayer growth may impact ensemble surface analytical measurements and should be considered when comparing measurements.

### 3.3. MHDA Monolayer and Cu-Ligated MHDA Multilayers Characterized Using In Situ Dynamic Spectroscopic Ellipsometry

**Figure 4:**
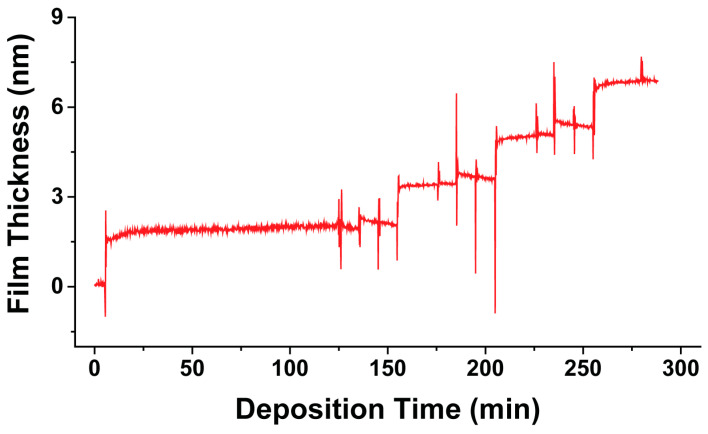
In situ dynamic spectroscopic ellipsometry data displaying the film thickness of the alternating-layer assembly of a Cu-ligated MHDA tetralayer as a function of time.

To complement the local morphology and structure of MHDA monolayers and Cu-ligated MHDA multilayers, in situ dynamic spectroscopic ellipsometry follows the assembly of a Cu-ligated MHDA multilayer. Figure 4 shows the film thickness as a function of deposition time for the assembly of a Cu-ligated MHDA multilayer. After ethanol is flushed through the liquid cell, the film thickness measures 0.0 ± 0.1 nm and reflects a clean and unfunctionalized Au{111} substrate. To form a MHDA monolayer, 15 mL of a 0.01 mM MHDA and 1.5 M acetic acid ethanolic solution is flushed through the liquid cell. Initially, the film thickness measures 1.6 ± 0.1 nm and gradually increases to 2.0 ± 0.1 nm over the course of 120 min. The increase in film thickness is consistent with the assembly of MHDA monolayers [17,20,21,22,29,31]. After the liquid cell is flushed with 15 mL of ethanol, the film thickness of the MHDA monolayer measures 2.0 ± 0.1 nm, which is consistent previous theoretical and experimental values of MHDA monolayers [17,20,21,22,29,31].

Subsequently, when the liquid cell with the MHDA monolayer is flushed with a 15 mL of a 5 mM Cu(ClO_4_)_2_·6H_2_O ethanolic solution, the film thickness initially increases to 2.3 ± 0.1 nm and then decreases to 2.1 ± 0.1 nm. This slight decrease in film thickness suggests that adsorption of the Cu(II) ions may impact the structure of the chemical film. When the liquid cell is flushed with 15 mL of ethanol, the chemical film measures 2.1 ± 0.1 nm. A Cu-ligated MHDA bilayer is formed when 15 mL of a 1 mM MHDA solution is flushed through the liquid cell. The film thickness initially measures 3.3 ± 0.1 nm and increases to 3.4 ± 0.1 nm. When the liquid cell is flushed with 15 mL of ethanol, the thickness measures 3.4 ± 0.1 nm. Then, the liquid cell with the Cu-ligated MHDA bilayer is flushed with 15 mL of a 5 mM Cu(ClO_4_)_2_·6H_2_O ethanolic solution, the film thickness increases to 3.8 ± 0.1 nm and then slowly decreases to 3.7 ± 0.1 nm. When the liquid cell is flushed with 15 mL of ethanol, the chemical film thickness measures 3.6 ± 0.1 nm. A Cu-ligated MHDA trilayer is formed when 15 mL of a 1 mM MHDA solution is flushed through the liquid cell. The film thickness initially measures 4.8 ± 0.1 nm and then slightly increases to 5.0 ± 0.1 nm. When the liquid cell is flushed with 15 mL of ethanol, the thickness measures 5.1 ± 0.1 nm. Subsequently, the liquid cell with the Cu-ligated MHDA trilayer is flushed with 15 mL of a 5 mM Cu(ClO_4_)_2_·6H_2_O ethanolic solution, the chemical film thickness initially increases to 5.5 ± 0.1 nm and then decreases 5.3 ± 0.1 nm. When the liquid cell is flushed with 15 mL of ethanol, the chemical film thickness measures 5.4 ± 0.1 nm. A Cu-ligated MHDA tetralayer is formed when 15 mL of a 1 mM MHDA solution is flushed through the liquid cell. The film thickness initially measures 6.7 ± 0.1 nm and then slightly increases to 6.9 ± 0.1 nm. When the liquid cell is flushed with 15 mL of ethanol, the thickness measures 6.9 ± 0.1 nm.

In general, the thicknesses of the Cu-ligated MHDA multilayer measured in ethanol after each deposition step (Table 1) are consistent with previous theoretical and ex situ experimental values [17,20,21,22]. However, in situ dynamic spectroscopic ellipsometry measurements provide a more comprehensive picture of the assembly process when compared to ex situ ellipsometry methods. For example, the thickness of Cu-ligated MHDA multilayers with Cu(II) ions increase upon exposure to the MHDA solution and then only slightly increase 0.1–0.3 nm over the next 20 min. Additionally, the thickness of the Cu-ligated MHDA multilayer increase upon exposure to a Cu(ClO_4_)_2_·6H_2_O ethanolic solution and then slightly decrease 0.1–0.2 nm over the next 10 min. This suggests that there are opportunities for the Cu-ligated MHDA multilayer assembly process to be optimized to minimize deposition times while having minimal impact to the quality of the chemical film.

The in situ dynamic spectroscopic ellipsometry measurements have several advantages over ex situ techniques. First, the same region of the substrate is being characterized, which helps mitigate fluctuations in the film thickness due to variations in the underlying substrates. Second, since the substrate remains fixed under the liquid cell, the same light path is measured throughout the experiment reducing positioning errors. Third, cross-contamination between the MHDA and Cu(ClO_4_)_2_·6H_2_O deposition steps is common during the deposition of the Cu-ligated MHDA multilayers due to incomplete rinsing. By flushing the 500 µL liquid cell with 15 mL of ethanol between each deposition step, this source of error is mitigated. Finally, the rinsing and drying of substrates between each deposition step to verify the successful deposition of a particular layer may have a profound impact on the overall structure of the Cu-ligated MHDA multilayer. By using in situ dynamic spectroscopic ellipsometry, there is no need to dry the substrate between deposition steps to characterize the resulting Cu-ligated MHDA multilayer.

## 4. Conclusions

The self-assembly of C18, C12, and MHDA monolayers and sequential layer-by-layer assembly of Cu-ligated MHDA multilayers have been investigated using in situ dynamic spectroscopic ellipsometry. The thicknesses of the chemical films agree with previous ex situ spectroscopic ellipsometry measurements and theoretical values. However, in situ dynamic spectroscopic ellipsometry provides insight into the assembly process without the need to interrupt the assembly process and potentially altering the characteristics of the chemical film. Furthermore, the real-time dynamics of the assembly of the monolayers and multilayers on Au{111} substrates can be followed, enabling their assembly to be optimized by minimizing their deposition. Efforts in utilizing in situ spectroscopic ellipsometry as a platform to optimize assembly conditions (deposition times, concentration, etc.) for Cu-ligated MHDA multilayers and to explore other metal-molecule multilayer systems are ongoing.

## Figures and Tables

**Table 1:** Chemical film thicknesses of a Cu-ligated MHDA multilayer measured in ethanol.

Layer (Deposition Time)	Layer Thickness in Ethanol (nm)
MHDA Monolayer (125–135 min)	2.0 ± 0.1
MHDA Monolayer + Cu (145–155 min)	2.1 ± 0.1
Cu-ligated MHDA Bilayer (175–185 min)	3.4 ± 0.1
Cu-ligated MHDA Bilayer + Cu (195–205 min)	3.6 ± 0.1
Cu-ligated MHDA Trilayer (225–235 min)	5.1 ± 0.1
Cu-ligated MHDA Trilayer + Cu (245–255 min)	5.4 ± 0.1
Cu-ligated MHDA Tetralayer (275–285 min)	6.9 ± 0.1

## Data Availability

The data presented in this study are available from the corresponding author upon reasonable request.
